# TRUB1 is a novel biomarker for promoting malignancy in colorectal cancer via NFκB signaling

**DOI:** 10.1093/gastro/goaf027

**Published:** 2025-04-21

**Authors:** Yingzhao Wang, Yonghuang Tan, Tianhao Zhang, Zhaoliang Wang, Jingru Gong, Zhenshuang Du, Yong Mei, Jinping Ma

**Affiliations:** Department of Gastrointestinal Surgery, The First Affiliated Hospital, Sun Yat-sen University, Guangzhou, Guangdong, P. R. China; Department of Thoracic Surgery, The First Affiliated Hospital, Sun Yat-sen University, Guangzhou, Guangdong, P. R. China; Department of Gastrointestinal Surgery, The First Affiliated Hospital, Sun Yat-sen University, Guangzhou, Guangdong, P. R. China; School of Modern Information Industry, Guangzhou College of Commerce, Guangzhou, Guangdong, P. R. China; School of Pharmaceutical Sciences, Southern Medical University, Guangzhou, Guangdong, P. R. China; Department of Gastrointestinal Surgery, Xiang’an Hospital of Xiamen University, School of Medicine, Xiamen University, Xiamen, Fujian, P. R. China; Department of Hepatobiliary Surgery, The Affiliated Hospital of Guizhou Medical University, Guiyang, Guizhou, P. R. China; Department of Gastrointestinal Surgery, The First Affiliated Hospital, Sun Yat-sen University, Guangzhou, Guangdong, P. R. China

**Keywords:** TRUB1, colorectal cancer, tumor biomarker, NFκB, BIRC3

## Abstract

**Background:**

Colorectal cancer (CRC) is one of the most aggressive malignancies of the digestive tract, characterized by aberrant post-transcriptional RNA modifications, including pseudouridine (Ψ). TruB pseudouridine synthase family member 1 (TRUB1) is a key pseudouridine synthase but its role in CRC progression remains unclear.

**Methods:**

Public databases and CRC cell lines were analysed to assess TRUB1 expression in CRC. Receiver-operating characteristic (ROC) curve analysis and survival analysis were performed to evaluate the diagnostic and prognostic significance of TRUB1. The impact of TRUB1 on tumor proliferation and Ψ modification was examined in TRUB1-knock-down HCT116 cell lines. Mechanistically, RNA sequencing of control and TRUB1-knock-down HCT116 cells was conducted to identify potential pathways, which were validated by using real-time polymerase chain reaction (PCR), Western blot, and immunofluorescence assays.

**Results:**

TRUB1 was significantly upregulated in CRC tumor tissues and cell lines. ROC analysis showed that TRUB1 had strong diagnostic potential and its overexpression was associated with poorer overall survival in CRC patients. In TRUB1-knock-down HCT116 cells, apoptosis increased and tumor growth slowed in nude mice, with a corresponding increase in apoptosis-related proteins and decreased Ψ modification. Mechanistically, RNA sequencing indicated that tumor necrosis factor α signaling via the nuclear factor kappa B (NFκB) pathway was activated in TRUB1-knock-down HCT116 cells. Further analysis identified Baculoviral inhibitor of apoptosis proteins repeat-containing 3 (BIRC3) as a potential downstream target gene that was regulated by TRUB1 in the NFκB pathway.

**Conclusions:**

TRUB1 serves as a potential biomarker for CRC diagnosis and prognosis, and it can inhibit apoptosis in CRC cells via BIRC3-mediated NFκB signaling.

## Introduction

Colorectal cancer (CRC) is a malignant gastrointestinal tumor that poses a significant threat to human life and health globally and is characterized by various genetic and epigenetic abnormalities [1]. According to the latest statistics, CRC ranks first in men and second in women in cancer-related deaths [2]. The 5-year survival rate for localized, non-metastatic CRC can reach 90%; however, when metastasis to nearby organs occurs, this rate drops to 72%. In cases of distant metastasis, the 5-year survival rate plummets to ∼15% [3]. While surgery, radiotherapy, chemotherapy, and targeted therapies have shown promising results in early and mid-stage CRC, effective treatments for advanced or metastatic CRC remain limited [4]. Thus, further research on biomarkers that aid in the diagnosis and treatment of CRC is crucial for improving patient survival and quality of life.

Pseudouridine (Ψ) modification is the most prevalent RNA modification and occurs in almost all types of RNA [5]. It is primarily catalysed by pseudouridine synthases (PUSs) and is stable, being detectable in urine, blood, and saliva [6]. The human genome encodes 13 PUSs [7], among which Pseudouridine synthase 7 (PUS7) and Dyskerin pseudouridine synthase 1 (DKC1) have been reported to be associated with CRC [8–10]. PUS7 has been shown to promote CRC cell metastasis and proliferation through the HSP90/PUS7/LASP1 signaling pathway or the Wnt/β–catenin pathway, although this process may be independent of PUS7’s Ψ modification activity [11]. Additionally, high DKC1 expression can promote CRC progression by increasing ribosomal protein expression through Ψ modification and interacting with the HRas proto-oncogene (HRAS) to inhibit the RAS/RAF/MEK/ERK signaling pathway [8, 10].

While DKC1 is a well-established CRC regulator, it belongs to the TRUB family, which also includes TruB pseudouridine synthase family member 1 (TRUB1) and TRUB2 [12]. The roles of TRUB1 and TRUB2 in CRC remain unclear. Though both are members of the same family, they perform distinct functions. TRUB1 is found in both the nucleus and cytoplasm, primarily catalysing Ψ modifications in nuclear tRNA. In contrast, TRUB2 is localized to the mitochondria, where it catalyses Ψ modifications of mitochondrial tRNA and mRNA, and promotes mitochondrial ribosome formation, thus playing a role in oxidative phosphorylation [12, 13]. Given that DKC1 can drive CRC proliferation by activating the RAS/RAF/MEK/ERK signaling pathway [10], which integrates surface receptor signals into nuclear pathways, we hypothesize that TRUB1 may similarly influence CRC development and prognosis through nuclear pathways.

In this study, we confirmed through public databases that TRUB1 is highly expressed in CRC patients and is associated with poor prognosis. Additionally, by comparing normal intestinal epithelial cells with CRC cells, we found that TRUB1 is broadly overexpressed in CRC cell lines. TRUB1 knock-down in HCT116 cells resulted in reduced proliferation and tumorigenic capacity both *in vivo* and *in vitro* with decreased Ψ modification. Mechanistically, we discovered that tumor necrosis factor α (TNFα) signaling via the nuclear factor kappa B (NFκB) pathway was activated in TRUB1-knock-down HCT116 cells, leading to a significant increase in apoptosis-related proteins. Furthermore, intersection analysis identified Baculoviral inhibitor of apoptosis proteins (IAP) repeat-containing 3 (*BIRC3*) as a key regulatory gene in this process, with elevated expression observed in TRUB1-knock-down HCT116 cells.

## Materials and methods

### Public data mining of TRUB1 expression

To analyse TRUB1 expression at both mRNA and protein levels, data from The Cancer Genome Atlas (TCGA) and the Clinical Proteomic Tumor Analysis Consortium (CPTAC) were utilized via the UALCAN platform (https://ualcan.path.uab.edu/). “TCGA_COAD” and “Proteomics Colon Cancer” datasets were selected for the analysis.

### Gene Expression Omnibus datasets processing

Expression data from Gene Expression Omnibus (GEO) datasets (GSE37182, GSE103512, GSE10905, GSE44076) were downloaded, including matrix and platform information, from the GEO database (http://www.ncbi.nlm.nih.gov/geo). All expression matrices were normalized and TRUB1 expression data were extracted for analysis. The expression and receiver-operating characteristic (ROC) analyses were performed by using the Bioinformatics platform (http://www.bioinformatics.com.cn).

### Survival analysis in Kaplan–Meier plotter

Survival analysis of TRUB1 in colon cancer was performed by using the Kaplan–Meier Plotter (http://kmplot.com/analysis/). TRUB1 (226339_at) expression data were analysed and patients were divided by using the “Auto select the best cut-off” option. After outlier removal, Kaplan–Meier survival curves for overall survival (OS), post-progression survival (PPS), and relapse-free survival (RFS) were generated. The GSE17538 dataset (*n *=* *232) was also used for survival analysis.

### Gene Set Enrichment Analysis

Gene Set Enrichment Analysis (GSEA) was conducted by using RNA-sequencing (RNA-seq) data from HCT116 control cells and TRUB1-knock-down HCT116 cells (shTRUB1-1, shTRUB1-2). Analysis was performed by using hallmark gene sets, with *P *<* *0.05 considered statistically significant.

### Cell culture and cell transfection

The NCM460, HCT116, SW480, SW620, and RKO cell lines were purchased from the American Type Culture Collection (ATCC; Manassas, VA, USA). Cells were cultured and maintained in Dulbecco’s Modified Eagle’s Medium (DMEM; #D1145; Merck, Rahway, NJ, USA) supplemented with 10% fetal bovine serum (FBS; #A5256701; Gibco, Waltham, MA, USA), 100 U/mL of penicillin and 100 µg/mL of streptomycin (#V900929; Merck). Cells were incubated in a humidified chamber at 37°C with 5% CO_2_. The TRUB1-shRNA (#GIEL0275918; GENECHEM, Shanghai, China) was transfection when cells had grown to 50%–60% according to the protocol. Subsequent experiments were performed 1 month after screening with puromycin (#A1113802; Merck).

### Mice and housing conditions

BALB/c-nu mice, aged 7 weeks, were obtained from the Laboratory Animal Center of Sun Yat-sen University. Animal experiments were approved by the Institutional Animal Care and Use Committee of Sun Yat-sen University (Approval No. SYSU-IACUC-2024–000154). The mice were housed under specific-pathogen-free conditions with a 12:12 light–dark cycle.

### Subcutaneous tumor model

HCT116 cells were digested into single cells by using 0.25% trypsin (#T2600000; Merck) and washed with cold phosphate buffered saline (PBS) thrice. BALB/c-nu mice were implanted with 2 × 10^6^ cells in 50 μL of PBS subcutaneously into the right flank on day 1. Beginning from day 7, tumor volumes of the mice were recorded by using vernier calipers and calculated by using (length × width × height)/2 [14]. On day 31, all tumor-bearing mice were sacrificed and tumors were isolated for weighing.

### Apoptosis assay

HCT116 cell lines were inoculated on six-well plates (#3516; CORNING, Glendale, AZ, USA). When the growth density reached ∼75%, cells were harvested and the apoptosis test was performed by using the AnnexinV, FITC Apoptosis Detection Kit (#Lot.SH680; DOJINDO, Kumamoto Ken, Kyushu, Japan). The ratio of Q2 quadrant was identified as apoptosis cells. The apoptosis assay was conducted for three individual repeats.

### RNA sequencing

RNA preparation, library construction, and sequencing were conducted on the BGISEQ platform (#BGISEQ-500; Huada, Shenzhen, Guangdong, China) at the ORIGIN-GENE (OriGene Technologies, Wuxi, Jiangsu, China). Different expressed genes with a fold change of ≥1.5 and a false discovery rate (FDR) of ≤0.05 were identified. The expression matrix was used for subsequent analysis. The matrix is shown in Supplementary Tables l and 2.

### Real-time polymerase chain reaction

RNA of HCT116 was extracted by using the RNAdvance Cell v2 kit (#A47942; Beckman, Indianapolis, IN, USA). For the quantitative PCR (qPCR)-based mRNA export analysis, all the reactions were performed by using a Takara SYBR Premix Ex Taq (#639676; Takara, San Jose, CA, USA) according to the manufacturer’s instructions and quantified by using a CFX96 Real-Time PCR System (Bio-Rad, Hercules, CA, USA). The relative fold changes were calculated by using the the 2(-Delta Delta C (T)) method. The primer pairs that were used for qPCR in this study are listed in Supplementary Table 3.

### Western blot analysis

The antibodies that were used in the Western blot were TRUB1 (1:2,000; #PA536003; Thermo, Waltham, MA, USA); TRUB2 (1:2,000; #K110029P; Solarbio, Beijing, China); p65 (1:2,000; #a22331; ABclonal, Woburn, MA, USA); phosphorylated-P65 (pP65; 1:2,000; #af2006; Affinity, Nottingham, UK); GAPDH (1:1,000; #BM3876; BOSTER, Pleasanton, CA, USA); Cytochrome C (1:2,000; #A13430; ABclonal); Bcl 2 (1:2,000; #A0208; ABclonal); Caspase-8 (1:2,000; #A0215; ABclonal); Caspase-3 (1:2,000; #A02156; ABclonal); Caspase-9 (1:2,000; #A11910; ABclonal); cleaved Caspase-3 (1:2,000; #9661 T; CST, Danvers, MA, USA); and β-Actin (1:2,000; #AC038; ABclonal). The supernatant containing 20 μg of proteins was analysed by using sodium dodecyl sulfate–polyacrylamide gel electrophoresis (SDS-PAGE). The proteins were then transferred onto the nitrocellulose membrane, which was further blocked with non-fat milk dissolved in PBS with 0.1% Tween-20 (PBST; #P1033; Solarbio) buffer. The primary antibodies were subsequently incubated with the nitrocellulose membrane overnight at 4°C. The next day, the nitrocellulose membrane was washed with PBST and subsequently incubated with an horseradish peroxidase (HRP)-conjugated secondary antibody at a dilution of 5,000 at room temperature for 1 h. The protein bands were visualized by using an enhanced chemiluminescence (ECL) and chemiluminescence instrument (GE Amersham Imager 600; Cytiva, Marlborough, MA, USA). The luminescent solution was incubated for 2 min and imaged by using a chemiluminescence meter.

### Dot plot assay

RNA of HCT116 was extracted by using a RNAdvance Cell v2 kit (#A47942; Beckman). The quantified RNA was serially diluted to concentrations of 800, 400, 200, 100, and 50 ng. The samples were then subjected to denaturation in a water bath at 55°C for 5 min. After denaturation, the RNA samples were immediately placed on ice. A nylon membrane of appropriate size was cut and fixed onto a flat surface by using tape at the corners. The serially diluted, denatured RNA samples were sequentially spotted onto the nylon membrane and allowed to air dry. Once dried, the membrane was subjected to ultraviolet (UV) crosslink (E1666; Xinzhi, Ningbo, China) at 1,200 µJ, 254 nm for 30 min. The cross-linked membrane was blocked with 5% non-fat dry milk for 1 h. After blocking, the membrane was gently washed three times with 1× PBST for 5 min each. The membrane was then incubated with the Pseudouridine Monoclonal antibody (#68578; Proteintech, Rosemont, IL, USA) at 4°C overnight. Following Pseudouridine Monoclonal antibody incubation, the membrane was quickly washed three times with 1× PBST for 10 min each and the Pseudouridine Monoclonal antibody was recovered. The membrane was subsequently incubated with the HRP-goat anti-mouse secondary antibody (#AP308P; Merck) for 1 h, after which the secondary antibody was also recovered. The membrane was washed five times with 1× PBST for 5 min each. After a 5-min incubation in SignalBright Max Chemiluminescent Substrate (#PK10013; Proteintech), the membrane was developed and photographed by using a chemiluminescence imaging system (ChemiDoc Go; Bio-Rad).

### Immunofluorescence assay

In total, 2 × 10^4^ control cells and 2 × 10^4^ shTRUB1 HCT116 cells were cultured in 24-well plates and incubated at 37°C with 5% CO_2_ for 36 h. Then, the adherent cells were washed thrice by using PBS and subsequently fixed with 4% paraformaldehyde for 20 min. Then, the cells were immersed in 0.5% Triton X-100 solution for 20 min at room temperature, after which the cells were incubated with 5% goat serum for 30 min to mitigate non-specific binding. Next, the cells were incubated with the indicated primary antibodies (anti-Caspase-9, 1:200, #A11910, ABclonal; anti-Caspase-3, 1:200, #A2156, ABclonal) at 4°C for 12 h. After the primary antibody incubation, the cells were treated with secondary antibodies (Dylight 488; #A23220; Abbkine, Wuhan, China) and incubated for 1 h. Finally, the cell nuclei were stained with DAPI (#RM02978; ABclonal) for 15 min at room temperature. Cells were imaged by using a confocal laser scanning microscope (FV3000; Olympus Corporation, Tokyo, Japan).

### Statistical analysis

Data were presented as mean ± standard error of the mean. Differences between two groups were analysed by using the Student’s *t*-test, while one-way analysis of variance (ANOVA) was used for comparisons between three or more groups. Pearson correlation was used for correlation analysis. *P *<* *0.05 was considered statistically significant.

## Results

### TRUB1 expression is upregulated in CRC

**Figure 1.**
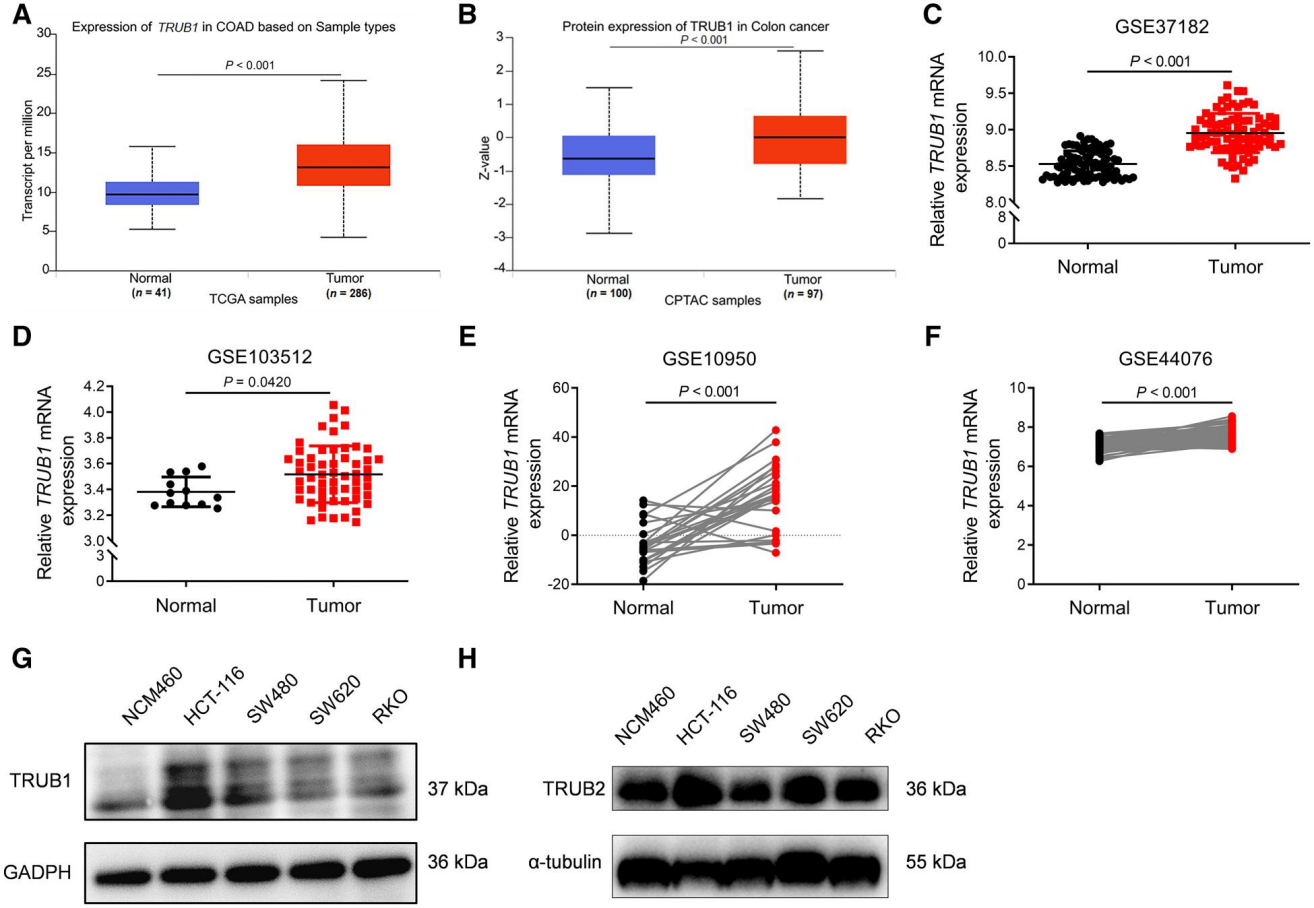
Expression of TRUB1 in CRC. (A) TRUB1 mRNA expression levels in normal and tumor tissues, based on the TCGA COAD database; (B) TRUB1 protein expression levels in normal and tumor tissues, based on the CPTAC-colon cancer database; (C, D) TRUB1 mRNA expression levels from the GEO database (GSE37182 and GSE103512) in normal and tumor tissues; (E, F) TRUB1 mRNA expression levels in matched normal and tumor tissues from the GEO database (GSE37182 and GSE103512); (G) TRUB1 expression in normal colonic epithelial cells (NCM460) and colon cancer cell lines (HCT116, SW480, SW620, and RKO); (H) TRUB2 expression in normal colonic epithelial cells (NCM460) and colon cancer cell lines (HCT116, SW480, SW620, and RKO). CRC = colorectal cancer, COAD = colorectal adenocarcinoma, TCGA = The Cancer Genome Atlas, CPTAC = Clinical Proteomic Tumor Analysis Consortium, GEO = Gene Expression Omnibus.

To investigate the expression of TRUB1 in CRC tissues and normal colorectal epithelial tissues, we analysed data from multiple public databases, including TCGA, CPTAC, and GEO. RNA-seq data from 41 normal and 286 primary CRC tissues were analysed for their transcript-per-million values. The results demonstrated a significant upregulation of TRUB1 in primary colorectal adenocarcinoma (COAD) tissues compared with normal tissues (Figure 1A). Protein-level analysis by using CPTAC data from 100 normal and 97 CRC tissues further confirmed elevated TRUB1 expression in CRC (Figure 1B). We also validated these findings by using GEO datasets (GSE37182 and GSE103512), in which the expression of TRUB1 was consistently higher in COAD tumor tissues than in normal tissues (Figure 1C and D). Additionally, we analysed paired tumor and normal tissue data from CRC patients in the GSE10950 and GSE44076 datasets. The results showed that TRUB1 expression remained significantly higher in tumor tissues compared with normal tissues in the paired data (Figure 1E and F), further confirming the specific overexpression of TRUB1 in CRC tissues.

We extended our investigation to the expression of TRUB1 and TRUB2 in CRC cell lines. Using NCM460 (a normal intestinal epithelial cell line) and four CRC cell lines (HCT116, SW480, SW620, and RKO), we found that TRUB1 was significantly upregulated in CRC cells at the protein level (Figure 1G). In contrast, TRUB2 showed only slight overexpression in HCT116, SW620, and RKO (Figure 1H). These results suggested that TRUB1 may play a more substantial role in CRC progression than TRUB2 and, thus, our subsequent research focused primarily on TRUB1. In summary, the combined data from public databases and protein expression analysis in cell lines indicate that TRUB1 expression is consistently higher in CRC tissues.

### TRUB1 as a biomarker for CRC diagnosis and prognosis

**Figure 2.**
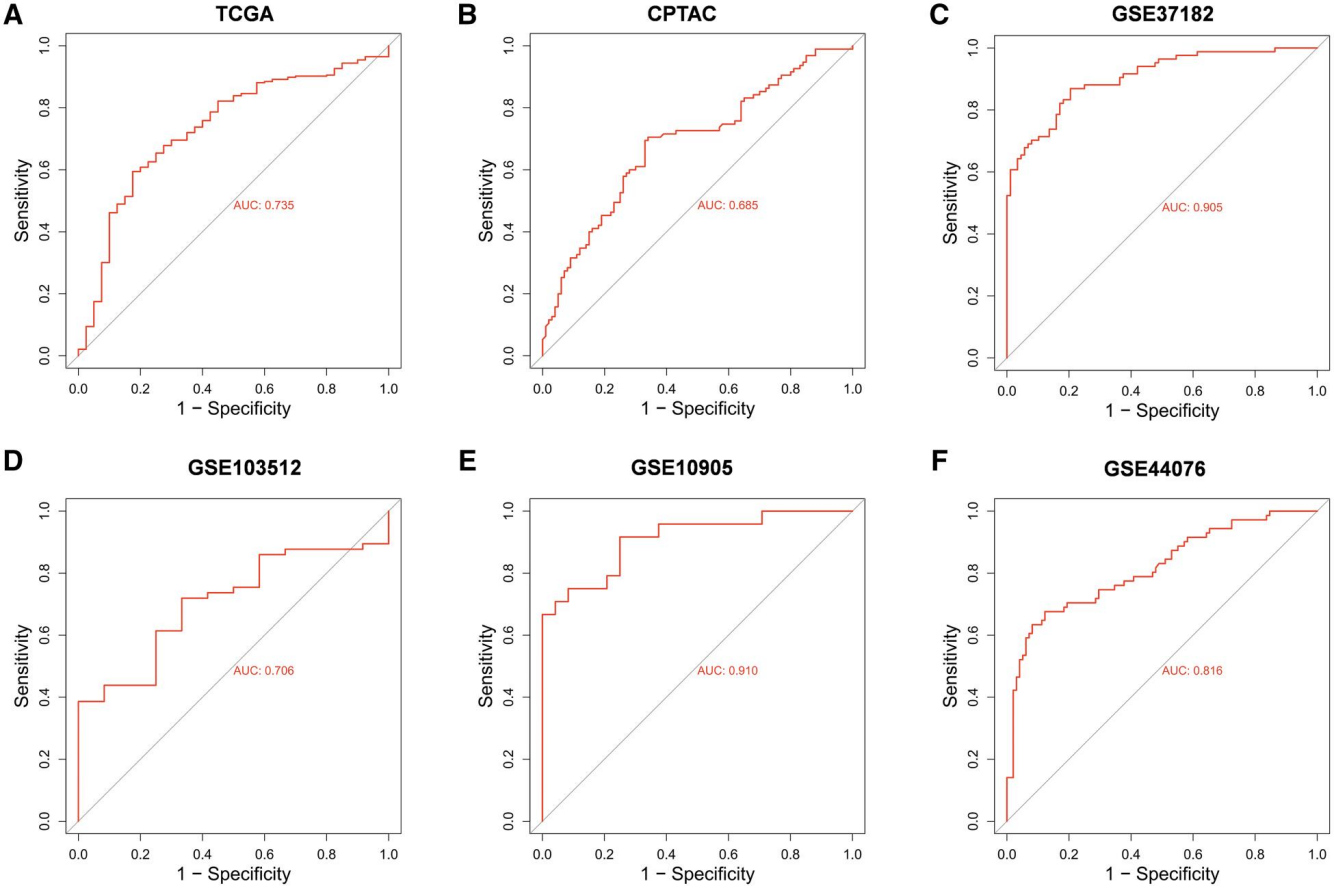
ROC analysis of TRUB1 in CRC based on multiple databases. ROC analysis of TRUB1 between normal and primary tumor tissues in CRC based on TCGA, CPTAC, and GEO datasets (GSE37182, GSE103512, GSE10905, and GSE44076). CRC = colorectal cancer, TCGA = The Cancer Genome Atlas, CPTAC = Clinical Proteomic Tumor Analysis Consortium, GEO Gene Expression Omnibus.

To further evaluate the diagnostic potential of TRUB1 in CRC, we conducted ROC curve analyses by using public datasets. The area under the curve (AUC) values were calculated for each dataset, revealing the following: AUCs of 0.735 for TCGA COAD, 0.685 for CPTAC, and 0.905 for GSE37182 (Figure 2A–C). Additionally, AUCs for GSE103512, GSE10905, and GSE44076 were 0.706, 0.910, and 0.816, respectively (Figure 2D–F). All AUC values exceeded 0.6, underscoring the potential of TRUB1 as a biomarker for distinguishing CRC patients from healthy individuals.

**Figure 3.**
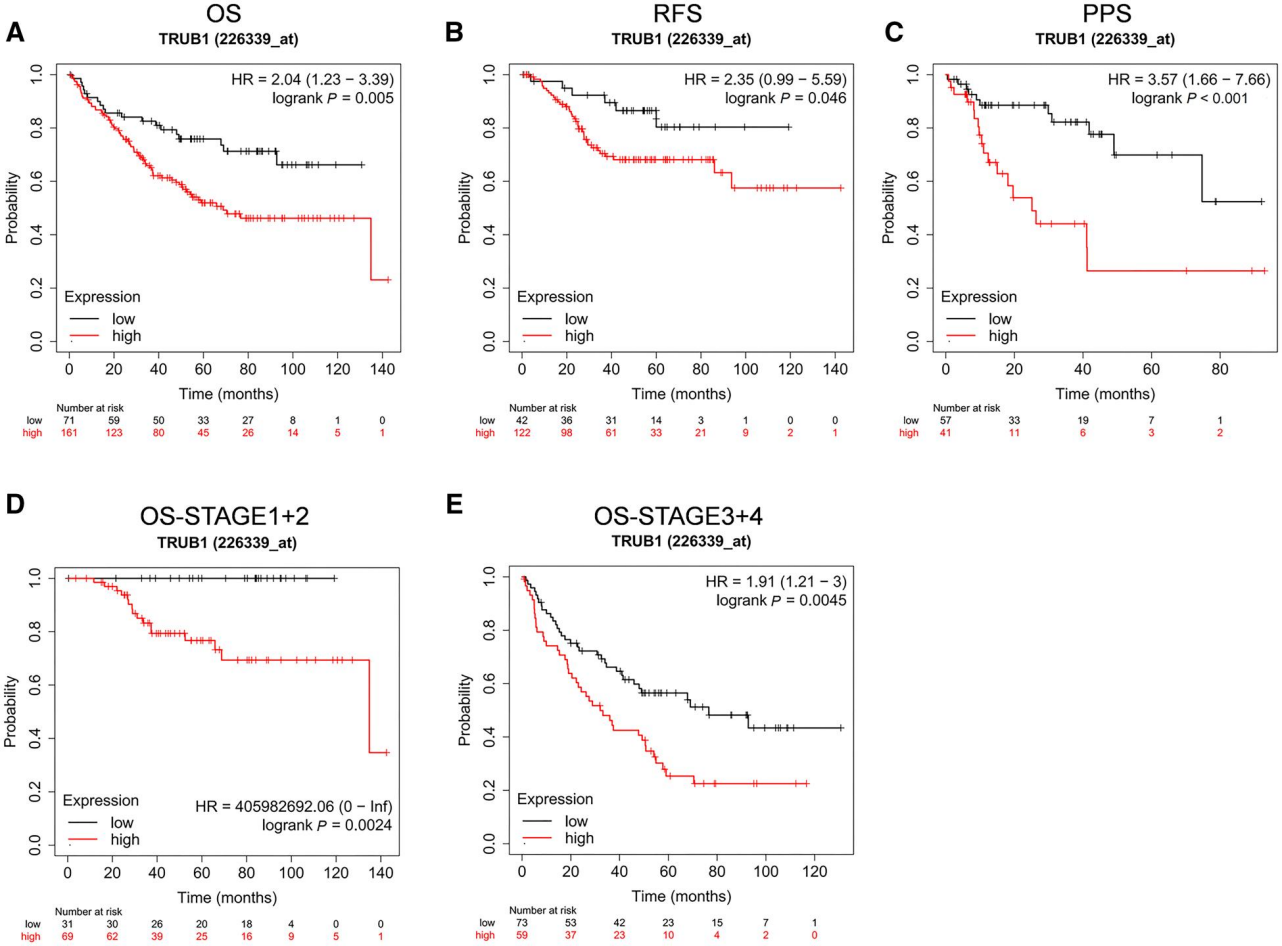
Impact of TRUB1 expression on CRC prognosis. (A) Kaplan–Meier OS curves comparing high and low TRUB1 expression in CRC based on GSE17538 (*n* = 232); (B) Kaplan–Meier RFS curves comparing high and low TRUB1 expression in CRC based on GSE17538 (*n* = 164); (C) Kaplan–Meier PPS curves comparing high and low TRUB1 expression in CRC based on GSE17538 (*n* = 98); (D, E) Kaplan–Meier OS curves comparing high and low TRUB1 expression in different CRC patients based on GSE17538 (*n* = 232). CRC = colorectal cancer, OS = overall survival, RFS = relapse-free survival, PPS = post-progression survival.

We also performed survival analyses to assess the impact of TRUB1 expression on CRC prognosis. Patients with higher TRUB1 expression exhibited shorter OS time (Figure 3A). Additionally, RFS and PPS analyses demonstrated that lower TRUB1 expression was associated with improved antitumor activity and better prognosis (Figure 3B and C). Further analysis across different clinical stages revealed that patients with elevated TRUB1 expression in stages 1 + 2 and 3 + 4 had shorter OS times, indicating a poorer prognosis (Figure 3D and E).

In conclusion, our results demonstrate that TRUB1 is not only upregulated in CRC, but also serves as a promising biomarker for both CRC identification and prognosis, with higher expression levels associated with worse clinical outcomes.

### Downregulation of TRUB1 suppresses tumorigenicity *in vivo* and *in vitro* with decreased Ψ modification

**Figure 4.**
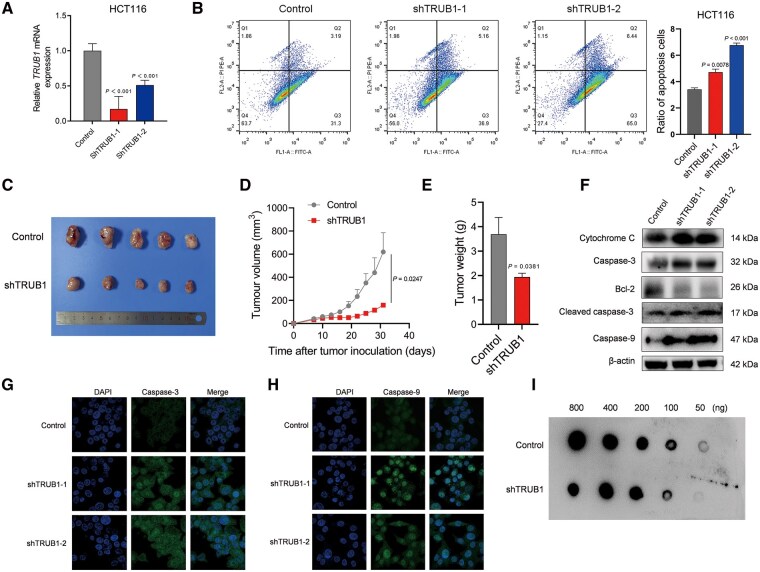
TRUB1 knock-down inhibits HCT116 proliferation and tumorigenesis with decreased Ψ modification. (A) RT-PCR results showing TRUB1 knock-down in HCT116 cells; (B) scatter plots and histograms showing apoptosis assay results for control and TRUB1-knock-down HCT116 cells; (C) photograph of tumors from control and TRUB1-knock-down HCT116 cells in BALB/c nude mice; (D) tumor weight in control and TRUB1-knock-down HCT116 cells in BALB/c nude mice; (E) tumor volume curves in control and TRUB1-knock-down HCT116 cells in BALB/c nude mice; (F) Western blot results of apoptosis-related proteins (Cytochrome C, Caspase-3, Bcl-2, cleaved Caspase-3, and Caspase-9) in control and TRUB1-knock-down HCT116 cells; (G) immunofluorescence assay results for Caspase-3 in control and TRUB1-knock-down HCT116 cells; (H) immunofluorescence assay results for Caspase-9 in control and TRUB1-knock-down HCT116 cells; (I) Ψ modification levels in control and TRUB1-knock-down HCT116 cells. Ψ = pseudouridine, RT-PCR = real-time PCR.

Given the association between TRUB1 overexpression and poor CRC prognosis, we sought to explore the potential mechanisms by which TRUB1 influences CRC progression. As HCT116 cells exhibited the highest TRUB1 expression among the CRC cell lines (Figure 1G), we generated stable TRUB1-knock-down HCT116 cells by using shRNA. Knock-down efficiency was confirmed via real-time PCR (RT-PCR) (Figure 4A). Next, the apoptosis results revealed that TRUB1 knock-down significantly promoted apoptosis in HCT116 cells (Figure 4B), suggesting that downregulation of TRUB1 hampers CRC cell proliferation.

To evaluate the effects *in vivo*, we used 10 BALB/c nude mice that were divided into two groups. Control and TRUB1-knock-down HCT116 cells (a mixture of shTRUB1-1 and shTRUB1-2) were subcutaneously injected into the mice. Tumor volume and images demonstrated that the downregulation of TRUB1 significantly reduced tumor growth in the TRUB1-knock-down group (Figure 4C and D). Tumor weights were also markedly lower in the TRUB1-knock-down mice (Figure 4E).

We further investigated the expression of apoptosis-related proteins in TRUB1-knock-down HCT116 cells by using Western blot and immunofluorescence assays. Western blot analysis showed that, following TRUB1 knock-down, Cytochrome C, Caspase-3, cleaved Caspase-3, and Caspase-9 were upregulated, whereas Bcl-2 was downregulated (Figure 4F). Immunofluorescence assays confirmed that Caspase-3 and Caspase-9 exhibited higher fluorescence intensity in TRUB1-knock-down cells (Figure 4G and H). These findings indicate that TRUB1 downregulation induces apoptosis in CRC cells *in vitro*.

As TRUB1 catalyses the Ψ modification of tRNA, we investigated whether TRUB1 downregulation affects Ψ modification levels in HCT116 cells. The results showed decreased Ψ modification in the TRUB1-knock-down group (Figure 4I). Collectively, these findings suggest that TRUB1 downregulation enhances apoptosis in CRC cells, suppresses tumorigenicity *in vivo*, and decreases Ψ modification.

### *BIRC3* is a crucial downstream gene regulated by TRUB1 in CRC malignant progression

**Figure 5.**
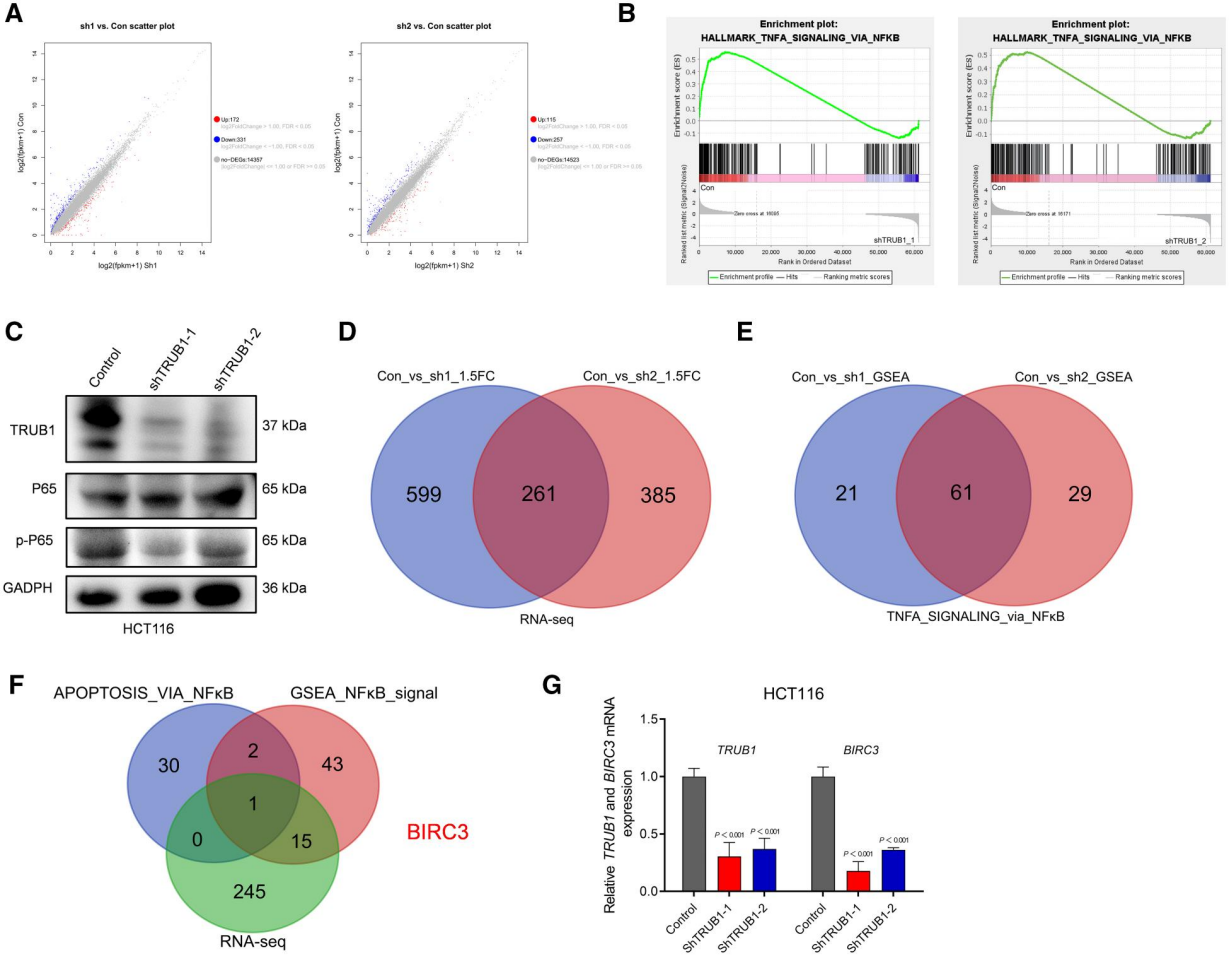
Potential downstream targets of TRUB1 in CRC. (A) Scatter plot showing genes with significant changes in RNA-seq after TRUB1 knock-down; (B) GSEA analysis showing enrichment of TNFα signaling via NFκB in cells with high TRUB1 expression; (C) Western blot results for TRUB1, P65, and pP65 in control and TRUB1-knock-down HCT116 cells; (D) intersection analysis of gene sets between HCT116-sh-TRUB1-1 and HCT116-sh-TRUB1-2 (FC ≥ 1.5, FDR ≤ 0.05); (E) intersection analysis of TNFα signaling via NFκB gene sets between HCT116-sh-TRUB1-1 and HCT116-sh-TRUB1-2 (*P* ≤ 0.05); (F) intersection analysis of gene sets between GSEA_NFκB signaling (61 genes), RNA-seq (261 genes), and apoptosis via NFκB (33 genes); (G) expression of TRUB1 and BIRC3 mRNA in control and TRUB1-knock-down HCT116 cells. GSEA = Gene Set Enrichment Analysis, pP65 = phosphorylated P65, BIRC3 = Baculoviral IAP repeat-containing protein 3.

To uncover the molecular changes induced by TRUB1 knock-down and identify potential downstream regulatory genes, we performed RNA sequencing on control and TRUB1-knock-down HCT116 cells. The volcano plots highlighted numerous differentially expressed genes in both shTRUB1-1 and shTRUB1-2 knock-down cell lines (Figure 5A). GSEA further indicated that TNFα signaling via NFκB was inhibited in the TRUB1-knock-down group (Figure 5B). To validate the effect of TRUB1 on the NFκB pathway, we conducted Western blot analysis to examine TRUB1, P65, and pP65 expression in TRUB1-knock-down HCT116 cells. While P65 levels remained unchanged, pP65 expression was notably reduced, suggesting that TRUB1 modulates NFκB signaling by inhibiting P65 phosphorylation (Figure 5C).

Next, we aimed to identify specific downstream genes that were regulated by TRUB1 within the NFκB pathway. By intersecting RNA-seq data (Figure 5D; 1.5-fold change, 261 genes), NFκB-signaling-related genes (Figure 5E; GSEA analysis, 61 genes), and apoptosis-related genes via NFκB (33 genes), we identified *BIRC3* as a potential downstream target of TRUB1 (Figure 5F and Supplementary Table 4). RT-PCR analysis confirmed that BIRC3 mRNA expression was significantly reduced following TRUB1 knock-down. In conclusion, our findings suggest that TRUB1 downregulation inhibits the NFκB pathway by reducing BIRC3 expression, thereby impairing CRC progression.

## Discussion

Previous studies have shown elevated urinary Ψ levels in CRC patients, suggesting its potential as a diagnostic biomarker for tumors [15]. The abnormal expression of PUSs, which catalyse Ψ modification, may underlie the increased Ψ levels that were observed in CRC patients [16]. In alignment with these findings, our study demonstrates that TRUB1 is highly expressed in CRC tissues and cell lines. ROC curve analysis yielded AUC values of >0.650 for TRUB1 as a tumor marker and CRC patients with high TRUB1 expression exhibited poorer OS, RFS, and PPS. These findings suggest that, in addition to DKC1 and PUS7 [8–10], which have already been implicated in CRC prognosis, TRUB1 is also a valuable prognostic marker.

Various PUSs, including PUS7, PUS1, and DKC1, have been shown to regulate tumorigenesis by modulating tumor cell proliferation and apoptosis [10, 11, 17, 18]. For instance, PUS7-dependent pseudouridylation of ALKBH3 mRNA enhances its translation efficiency, inhibiting gastric cancer cell proliferation and tumor growth [19]. Similarly, PUS7 knockout in glioma stem cells (GSCs) increases the expression of interferon-stimulated genes and inhibits GSC growth [17]. PUS1 is involved in several tumor-related processes, such as mitophagy and the PI3K–Akt signaling pathway [20]. PUS1 knock-down has been shown to inhibit hepatocellular carcinoma (HCC) cell proliferation and tumorigenesis, whereas PUS1 overexpression promotes HCC growth both *in vitro* and *in vivo* [18]. DKC1 has been linked to malignant proliferation and invasion in prostate cancer, glioma, and CRC [8, 21]. In parallel, our research revealed that TRUB1 knock-down significantly inhibited HCT116 cell proliferation both *in vitro* and *in vivo*, accompanied by increased expression of apoptosis-related proteins, which aligns with the mechanisms observed for other PUSs. Furthermore, as tumors exhibit elevated Ψ modification levels, patients with cancer often show higher blood and urine Ψ concentrations [22]. Several studies suggest that Ψ could serve as a reliable biomarker for various cancers, including gastric [19], liver [23], and lung [24]. Notably, DKC1 knock-down or inhibition has been shown to reduce Ψ levels in CRC cells [10]. Likewise, our experiments demonstrated that TRUB1 knock-down reduced Ψ levels in HCT116 cells, indicating that TRUB1 might influence CRC progression by modulating Ψ synthesis.

To further investigate the mechanisms through which TRUB1 impacts CRC proliferation, we performed RNA sequencing on control and TRUB1-knock-down HCT116 cells. GSEA revealed that TNFα signaling via NFκB was significantly inhibited in TRUB1-knock-down cells. The NFκB transcription factor is a crucial regulator of tumor metastasis and growth, typically functioning as a heterodimer or homodimer of RelA/p65, RelB, p52, p50, and c-Rel [25]. TNFα, a pro-inflammatory cytokine, is a potent upstream activator of NFκB [26]. Upon TNFα stimulation, the TNF receptor forms a complex that activates IKKα/β, leading to the phosphorylation and degradation of IκB, thus freeing NFκB to translocate to the nucleus and regulate target genes [27]. Overactivation of the TNFα signaling via the NFκB pathway has been linked to tumor progression, with excessive activation driving tumor cell proliferation [26, 28]. Our study found that, although p65 expression remained unchanged in TRUB1-knock-down cells, pP65 was significantly reduced, indicating that TRUB1 knock-down inhibits NFκB pathway activation by suppressing p65 phosphorylation. This blockage of the NFκB pathway likely contributes to the reduced tumor growth that was observed in our experiments, consistently with previous reports.

Finally, through the integration of differentially expressed genes from RNA-sequencing data with NFκB signaling and apoptosis-related genes, we identified *BIRC3* as a key downstream target of TRUB1. RT-PCR confirmed the downregulation of BIRC3 in TRUB1-knock-down cells. BIRC3, a member of the IAP family, has been implicated in several cancers, including esophageal cancer [29], head and neck cancer [30], and CRC [31]. Elevated BIRC3 levels are associated with poor prognosis [32]. BIRC3 regulates caspase activation through its E3 ligase activity and its downregulation can promote apoptosis via the TNFα/NFκB signaling pathway [33]. Our findings that TRUB1 knock-down inhibits BIRC3 expression, along with the suppression of TNFα signaling via NFκB, underscore the role of BIRC3 in CRC tumor progression and suggest that TRUB1 may regulate CRC growth through BIRC3-dependent mechanisms.

Although our study sheds light on the function of TRUB1 in CRC, there are some limitations. First, given the extensive use of public datasets, there may be inherent biases in the analyses. Second, while we observed reduced Ψ modification following TRUB1 knock-down, further *in vivo* studies are needed to confirm the detailed effects of TRUB1 on Ψ modification in CRC. Third, while the molecular mechanisms of TRUB1-mediated regulation of BIRC3 were studied *in vitro*, more comprehensive *in vivo* experiments are necessary to validate these findings.

In conclusion, our study identifies TRUB1 as a potential prognostic biomarker for CRC. High TRUB1 expression is associated with poor prognosis and our experiments demonstrate that TRUB1 promotes tumorigenesis by inhibiting apoptosis. Mechanistically, we propose that the BIRC3-dependent TNFα signaling via the NFκB pathway is involved in TRUB1-mediated CRC progression. These findings suggest that TRUB1 could be a valuable therapeutic target for CRC treatment.

## Supplementary Material

goaf027_Supplementary_Data

## Data Availability

The datasets that were used in this study are publicly available. RNA-seq transcriptome data were obtained from The Cancer Genome Atlas (TCGA) (https://tcga-data.nci.nih.gov/tcga) and the Gene Expression Omnibus (GEO) database (https://www.ncbi.nlm.nih.gov/geoprofiles/? term).
